# Supplementary material for: The Poplar (Populus trichocarpa) Dehydrin Gene PtrDHN-3 Enhances Tolerance to Salt Stress in Arabidopsis
Source: Plants (Basel). 2022 Oct 13;11(20):2700. doi: 10.3390/plants11202700 (PMC9611832; doi:10.3390/plants11202700)
Supplement: Supplementary file 1 [file plants-11-02700-s001.zip › plants-1918305-SI.pdf]

Supplementary Materials

Supplementary Figure S1. PtrDHN-3 in Arabidopsis lines was confirmed by PCR

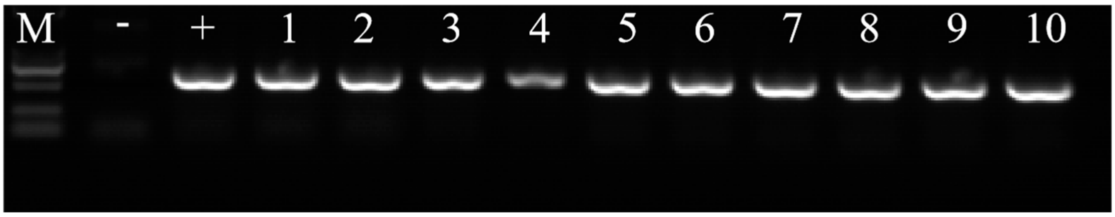

Supplementary Table S1. qRT-PCR primer sequence.

Name	Sequence(5'->3')
SOD1-F	CCCCGATGGTAAAACACACG
SOD1-R	CTTTCCGAGGTCATCAGGGT
SOD2-F	GTCTCTTGGG ACTTTCACGA
SOD2-R	TAAGCGAGCTTTTCGACCAG
SOD3-F	TCCAATCTGT TTCCTTCGCC
SOD3-R	CGTGTGTCATGTTGTTAGGG
POD1-F	TCTGTTTATT CTCGTATCTT
POD1-R	TCAACCATGCAATCGTGGAA
POD2-F	CTTTGGCGGC AACAAAAGGA
POD2-R	AGAGTTCTTCTCAGTAACTA
POD3-F	CTTTACCTCA TTTACGCTCT
POD3-R	CACAACCCTGAACGAAACAA
P5CS1-F	GCCAGAGACG TCAAACGTAT
P5CS1-R	TTGTCCAACACCAGCACAAG
P5CS2-F	TGGGAAAGGT GGAAGATTGG
P5CS2-R	GTCACATCCAACTGGTCAAA
AtActin2-F	CCCAGTGTTGTTGGTAGGCCAAGAC
AtActin2-R	CATAGCGGGAGAGTTAAAGGTCTC
PtrActin2-F	AACATGGGATTGTTAGCAACTGG
PtrActin2-R	TCCATCACCAGAATCCAGCACA
